# Supplementary material for: Individual differences in ethics positions: The EPQ-5
Source: PLoS One. 2021 Jun 21;16(6):e0251989. doi: 10.1371/journal.pone.0251989 (PMC8216522; doi:10.1371/journal.pone.0251989)
Supplement: S1 Table — (DOCX) [file pone.0251989.s001.docx]

# S1: The Short Ethics Position Questionnaire (EPQ-5)

You will find a series of general statements listed below. Each represents a commonly held opinion and there are no right or wrong answers. You will probably disagree with some items and agree with others. We are interested in the extent to which you agree or disagree with such matters of opinion.

Please read each statement carefully. Then indicate the extent to which you agree or disagree where:

1 = Strongly disagree 3 = Neutral 4 = Agree

2 = Disagree 5 = Strongly Agree

1. A person should make certain that their actions never intentionally harm another even to a small degree.
2. The existence of potential harm to others is always wrong, irrespective of the benefits to be gained.
3. One should never psychologically or physically harm another person.
4. One should not perform an action which might in any way threaten the dignity and welfare of another individual.
5. If an action could harm an innocent other, then it should not be done.
6. What is ethical varies from one situation and society to another.
7. Moral standards should be seen as being individualistic; what one person considers to be moral may be judged to be immoral by another person.
8. Questions of what is ethical for everyone can never be resolved since what is moral or immoral is up to the individual.
9. Moral standards are simply personal rules that indicate how a person should behave, and are not to be applied in making judgments of others.
10. Ethical considerations in interpersonal relations are so complex that individuals should be allowed to formulate their own individual codes.
