# Supplementary material for: Individual differences in ethics positions: The EPQ-5
Source: PLoS One. 2021 Jun 21;16(6):e0251989. doi: 10.1371/journal.pone.0251989 (PMC8216522; doi:10.1371/journal.pone.0251989)
Supplement: S2 Table — (DOCX) [file pone.0251989.s002.docx]

## S3: Study 3 Full Correlational Matrix for variables

|  | 1 | 2 |  | 3 | 4 | 5 | 6 | 7 | 8 | 9 | 10 | 11 | 12 | 13 | 14 |
| --- | --- | --- | --- | --- | --- | --- | --- | --- | --- | --- | --- | --- | --- | --- | --- |
| 1. Idealism | (.85) |  |  |  |  |  |  |  |  |  |  |  |  |  |  |
| 2. Relativism | .043^**^ | (.81) |  |  |  |  |  |  |  |  |  |  |  |  |  |
| 3. Sex | -.221^**^ | -.048^**^ |  | (-) |  |  |  |  |  |  |  |  |  |  |  |
| 4. Age | .066^**^ | -.157^**^ |  | -0.008 | (-) |  |  |  |  |  |  |  |  |  |  |
| 5. Harm | .562^**^ | .037^**^ |  | -.286^**^ | .113^**^ | (-.71) |  |  |  |  |  |  |  |  |  |
| 6. Fairness | .392^**^ | .064^**^ |  | -.115^**^ | .060^**^ | .545^**^ | (.67) |  |  |  |  |  |  |  |  |
| 7. Loyalty | -.086^**^ | -.138^**^ |  | .037^**^ | .054^**^ | -0.019 | -.115^**^ | (.72) |  |  |  |  |  |  |  |
| 8. Authority | -.105^**^ | -.209^**^ |  | 0.012 | .128^**^ | -.094^**^ | -.202^**^ | .677^**^ | (.76) |  |  |  |  |  |  |
| 9. Sanctity | .050^**^ | -.323^**^ |  | -.078^**^ | .096^**^ | .059^**^ | -.132^**^ | .556^**^ | .659^**^ | (.85) |  |  |  |  |  |
| 10. Self-Transcendence | .442^**^ | .035^*^ |  | -.193^**^ | .165^**^ | .553^**^ | .453^**^ | -0.014 | -.066^**^ | 0.025 | (.72) |  |  |  |  |
| 11. Conservation | .114^**^ | -.192^**^ |  | -.037^*^ | .196^**^ | .104^**^ | -0.016 | .483^**^ | .575^**^ | .574^**^ | .392^**^ | (.78) |  |  |  |
| 12. Openness | .073^**^ | .174^**^ |  | -.035^*^ | 0.012 | .062^**^ | .131^**^ | -.084^**^ | -.160^**^ | -.177^**^ | .375^**^ | .080^**^ | (.63) |  |  |
| 13. Self-enhancement | -.140^**^ | -0.018 |  | -0.002 | -.122^**^ | -.121^**^ | -.059^**^ | .241^**^ | .232^**^ | .134^**^ | .072^**^ | .324^**^ | .356^**^ | (.70) |  |
| 14. Hedonism | -0.014 | .230^**^ |  | .049^**^ | -.139^**^ | -0.006 | .073^**^ | -.072^**^ | -.133^**^ | -.235^**^ | .119^**^ | -0.018 | .409^**^ | .304^**^ | (-) |

Note: N = 8,638 for correlations between EPQ and MFQ, and N = 3,768 for correlations between EPQ and SVS.
