# Supplementary material for: Individual differences in ethics positions: The EPQ-5
Source: PLoS One. 2021 Jun 21;16(6):e0251989. doi: 10.1371/journal.pone.0251989 (PMC8216522; doi:10.1371/journal.pone.0251989)
Supplement: S1 Text — (DOCX) [file pone.0251989.s003.docx]

# S2: Supplemental Text of Measurement Invariance Tests

These materials will be posted at the *Center for Open Science* (COS), OSFramework. They are provided here for use during the review of this paper for possible publication.

## Model specifications in Study 1

Beginning with the one-factor solution presented in Table 2, we unsurprisingly found poor fit (χ^2^(170) = 5991, CFI = .542, RMSEA = .111 (.109; .113), SRMR = .113). We next moved on to the 2-factor model and fit was much improved, composite reliabilities (CR) were adequate for both factors (CR-Idealism = .81; CR-Relativism = .73), and the model achieved acceptable fit for the RMSEA and SRMR, but not the CFI (χ^2^(169) = 5991, Δχ^2^(1) = 3057, CFI = .782, RMSEA = .077 (.074; .079), SRMR = .070). However, the average variance extracted (AVE) was marginal for idealism and poor for relativism (AVE-Idealism = .32; AVE-Relativism = .23). Further, an examination of the standardized factor loadings revealed that eight items (four for Idealism, four for Relativism) were less than .40. In sum, support for the two-factor solution was mixed.

With mixed support for the overall model fit and a number of items failing to demonstrate acceptable psychometrics, we next considered an alternative specification proposed in the literature of a three factor solution that contains the veracity subscale consisting of the last two items of the relativism scale. This three factor model showed adequate fit in terms of the RMSEA and SRMR, but the CFI was just below Hu and Bentler’s suggested threshold of .90. In terms of the veracity items, the factor loading were acceptable (.81 & .80). However, both the idealism and relativism factors still contained several problematic items with low factor loadings and the marginal value of the CFI could be indicating the presence of important unmodelled crossloadings and/or correlated residuals. Our conclusion is “improved but in need of improvement.”

The three factor model demonstrated better results than the original two factor conceptualization, but we suggest the better psychometrics come with a hefty cost to theory. A three-factor EPQ scale is misaligned with EPT. EPT exclusively encompasses idealism and relativism with no mention of veracity. Rather than align the theory with the scale, we opted to align the scale with the theory and removed the two veracity items from the EPQ and re-ran the two-factor CFA. This is the fourth model presented in Table 2. Because this model is not nested with the others, there is no chi-square difference test, but the indices of global fit were very similar to the model that included veracity (CFI = .884, RMSEA = .058 (.055; .061), SRMR = .051), and CR and AVE were identical for idealism and nearly identical for relativism (CR-Relativism = .72; AVE-Relativism = .26). Although now theoretically aligned with EPT, there still is the issue of low factor loadings, low AVEs, and marginal overall model fit, at least for the CFI.

## Measurement invariance tests in Study 2

We conducted tests of measurement and structural invariance (MI/SI tests) to determine if the observed sex differences reflect underlying differences in the *constructs* of idealism and relativism or instead differences in the interpretation of the *measures*. Tests of MI assess if membership to one group precludes the ability to interpret the EPQ-5 the same way as members of the other group (Vandenberg & Lance, 2000). Tests of SI determine if there are group differences in the latent means or latent variances. These tests will determine if men and women perceive the EPQ-5 and their underlying constructs of idealism and relativism the same way and whether or not there are true sex differences in idealism and relativism.

Using procedures outlined in de Schoot, Lugtig, and Hox (2012) and Vandenberg and Lance (2000), configural, metric, scalar, residual variance invariance tests for MI and factor variance and factor mean invariance tests for SI were conducted in Mplus 8.0 (Muthén & Muthén, 1998). Classically, the χ^2^ statistic was used as the primary means of testing whether more restrictive models significantly worsened fit. However, when sample sizes are large such as those reported here, the χ^2^ can reject reasonable models with minimal misspecification (Kline, 2015). Thus, we opted for fit indices less influenced by sample size that nevertheless are capable of detecting misfit at levels to warrant concerns that the sexes view the EPQ-5 items differently. Consistent with past MI studies, we triangulated on the degree of MI with a comparative fit index (CFI) and two absolute fit indices (RMSEA and SRMR). We used Chen (2007) criterion of -.01 change in CFI paired with RMSEA increases of .015 and SRMR increases .030 for metric invariance and .015 for scalar and residual invariance.

We began with a configural invariance model where the EPQ-5 was tested simultaneously with all men’s and women’s specified paths allowed to vary (i.e., no between group constraints). As shown in Table 4, the configural model was a good fit (χ^2^(68) = 691, CFI = .982, RMSEA = .042 (.039; .045), SRMR = .038). These results allow us to test more restrictive models to determine the extent and form of MI and structural non-invariance.

A metric invariance test determines if factor loading differences exist between men and women. Again, as shown in Table 4, Chen’s (2007) criterion was met (ΔCFI = .002, ΔRMSEA = .000, ΔSRMR = .015) and Figure 1 shows the high similarity between men’s and women’s factor loadings on each item. The EPQ-5 shows metric invariance. Demonstrating scalar invariance for men and women would indicate that “the meaning of the construct (the factor loadings), and the levels of the underlying items (intercepts) are equal” (DeShoot et al., 2012). The equality constraint across both factor loadings and intercepts did not significantly worsen fit (ΔCFI = -.003, ΔRMSEA = .001, ΔSRMR = -.014). Our support of scalar invariance (i.e., “strong invariance”) indicates that the observed differences in item means between men and women is attributable to factor mean differences only. Finally with regard to MI, we tested for equality of the unstandardized residual variances to determine if unaccounted for variance in the idealism and relativism indicators significantly differed across men and women. The residual variance invariance test introduced ten equality constraints (error variances forced to be equal across sexes), but as shown in Table 4, the decrease in fit was not significantly worse than the scalar invariance results (ΔCFI = .009, ΔRMSEA = .005, ΔSRMR = .004), thus we retain the residual variance model. The retention of the residual variance invariance model, known as “strict invariance,” indicates that the amount of item variance not accounted for by idealism and relativism was the equivalent across men and women.

Having achieved MI, we then moved on to test for differences at the construct level. For the first SI test, we constrained men and women’s factor variances for idealism to be equal and men and women’s factor variance for relativism to be equal. These two constraints will determine if the amounts of individual differences in idealism and relativism is consistent across sexes. Once again, we see little change in the CFI and RMSEA (ΔCFI = .003, ΔRMSEA = .002), but there was a notable increase in the SRMR (ΔSRMR = .018). Given the collective evidence including the rather modest increase in χ^2^ and that all three global fit indices still met traditional thresholds of good fit, we retain this model, but note that men do have slightly more variance in idealism and relativism than women.

Finally, we constrained the factor means to determine if the sex differences found at scale level exist at the latent variable level after all constraints have been put in place. Constraining the factor means to equality resulted in the largest increases in misfit with the χ^2^ increasing over 500 points, and the most significant worsening of the global fit indices (ΔCFI = .015, ΔRMSEA = .009, ΔSRMR = .022). The CFI had borderline good fit at .950, the RMSEA and its confidence interval both fell above .050, and the SRMR exceeded .080. The significant increases in misfit and the overall model results led us to reject the full factor mean constrained model. However, the preponderance of misfit at the factor mean level was found with the idealism constraint. Thus, we supplemented this full factor mean invariance test with one where only the relativism factor mean was constrained. Doing so, resulted in a significant improvement over the Both Factor Mean model and showed nearly identical results to the Factor Variance model (ΔCFI = .001, ΔRMSEA = .000, ΔSRMR = .001). This means that men and women do not significantly differ in relativism, but do significantly differ in idealism. The mean relativism factor invariance model (bolded in Table 4) was retained and serves as our final model. The retention of this model and the prior MI results provide compelling evidence that men and women interpret the EPQ-5 items in similar ways, have similar levels of variance in both idealism and relativism, differ in their latent mean levels of idealism, but not relativism.

## Classification Analysis

The ethics position theory typology classifies people into one of four categories; it assumes individuals in a category share certain features in common and that those features distinguish them from other types of individuals. The moral types are qualitatively different from one another and, to some extent, mutually exclusive. However, ethics position theory is a modal multidimensional species type of classification system, as identified by Buss and Poley (1976). The idea of distinct ethics positions, although a logical derivation from the theory’s modal multidimensional specification of types, may not correspond to the natural groups that exist within the population. Rather than four categories, people may naturally cluster into fewer categories, more categories, or no categories at all.

A number of methods can be used to identify clusters of individuals within a more general population, including Bayesian expectation maximization algorithms, cluster analysis, latent class modeling, and taxonometric procedures (Waller & Meehl, 1998). In this study, we applied one of these methods, hierarchical cluster analysis (HCA), to classify a large group of individuals who had completed the EPQ. That analysis is presented in the manuscript, but to save space we did not include the chart of the magnitude of the squared Euclidean distances between the clusters in the final 12 steps in the cluster analysis. The elbow indicates the increase in the heterogeneity (distances) caused when empirically distinct clusters are combined in a single cluster.
