# Supplementary material for: Individual differences in ethics positions: The EPQ-5
Source: PLoS One. 2021 Jun 21;16(6):e0251989. doi: 10.1371/journal.pone.0251989 (PMC8216522; doi:10.1371/journal.pone.0251989)

### **S4: Study 3 MANCOVA Results for Values and Moral Foundations**

We also examined the relationship between the two scales of the EPQ and individuals’ values in two separate 2 (idealism: low and high) X 2 (relativism: low and high) X 2 (sex: men and women) MANOVAs—one for the SVS variables and one for the MFQ variables—with age as a covariate. These results are consistent with the correlational analyses presented in Table 6.

|  | Effect F-ratio (η_p_^2^) | | | | |
| --- | --- | --- | --- | --- | --- |
| Value | Age | Idealism | Relativism | Sex | Sex * Idealism * Relativism |
| Self-Transcendence | 79.36 (.024) | 414.68 (.116) | 1.84* (.001) | 57.15 (.018) | 7.60 (.002) |
| Conservation | 103.66 (.032) | 22.53 (.007) | 51.00 (.016) | 1.04* (.000) | 3.66* (.001) |
| Openness | 3.62* (.001) | 5.43 (.002) | 68.29 (.021) | 2.63* (.001) | 13.77 (.004) |
| Self-Enhancement | 45.36 (.014) | 50.41 (.016) | 4.29 (.001) | 0.91* (.000) | 3.53* (.001) |
| Hedonism | 44.05 (.014) | .001* (.000) | 79.55 (.025) | 5.45 (.002) | 4.99 (.002) |

* ns, all other effects *p* < .05


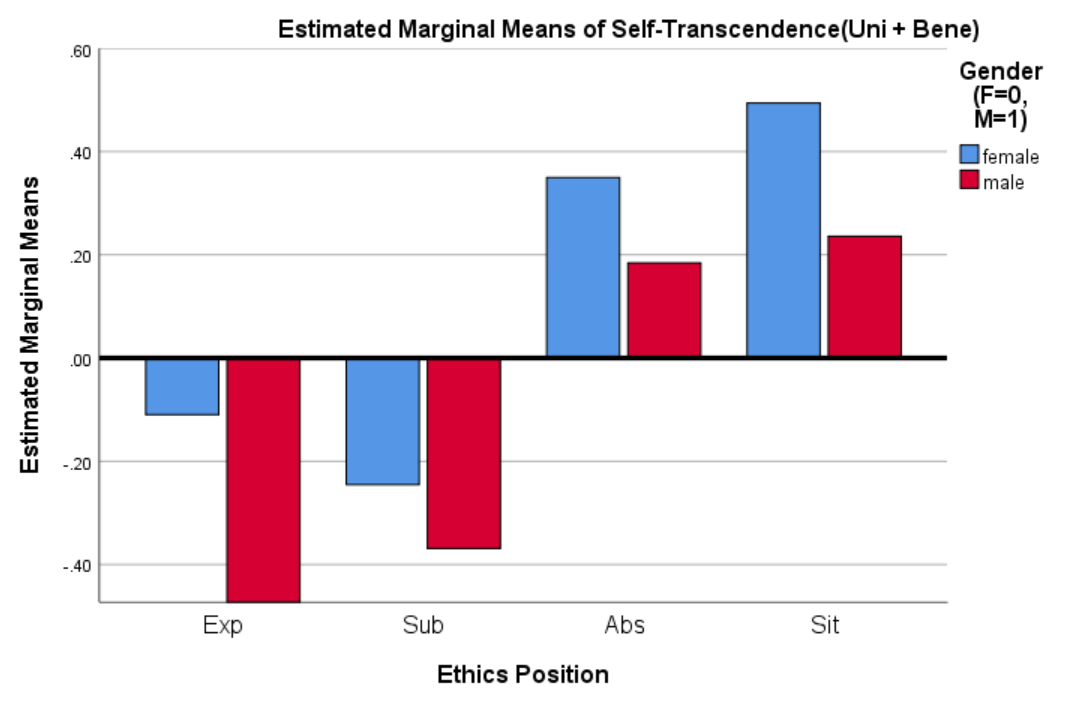


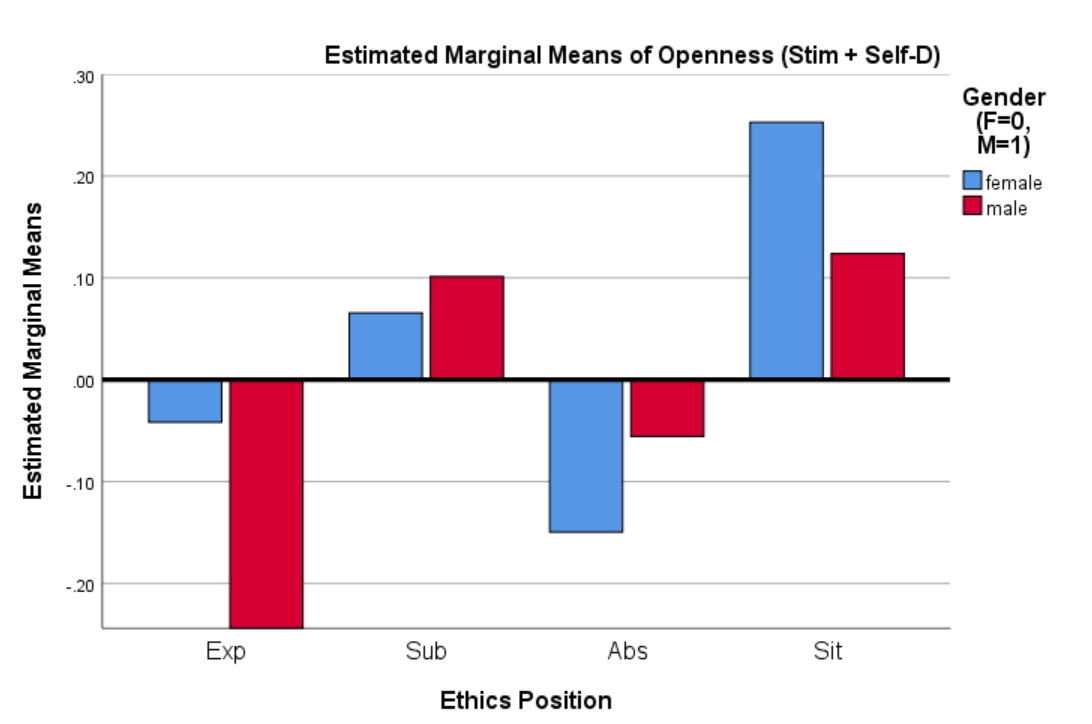


### MANCOVA for Moral Foundations

| Foundation | Effect F-ratio (η_p_^2^) | | | | | | |
| --- | --- | --- | --- | --- | --- | --- | --- |
|  | Age | Idealism | Relativism | Sex | Sex * Idealism | Sex * Relativism | Idealism * Relativism |
| Harm | 73.08 (.010) | 1767.84 (.195) | 1.66 (.000)* | 391.71 (.051) | 13.80 (.002) | 4.27 (.001) | 2.46 (.000)* |
| Fairness | 18.25 (.002) | 758.20 (.094) | 8.23 (.001) | 26.36 (.004) | 5.31 (.001) | 1.63 (.000)* | .47 (.000)* |
| Ingroup | 17.38 (.002) | 23.37 (.003) | 46.82 (.006) | 4.42 (.001) | 16.05 (.002) | 5.39 (.001) | 5.37 (.001) |
| Loyalty | 108.17 (.015) | 48.53 (.007) | 112.37 (.015) | .28 (.000)* | 12.51 (.002) | 19.69 (.003) | 6.73 (.001) |
| Sanctity | 43.10 (.005) | 13.81 (.002) | 413.87 (.054) | 48.15 (.007) | 10.64 (.001) | 8.12 (.001) | 12.97 (.002) |

* ns, all other effect *p* < .001


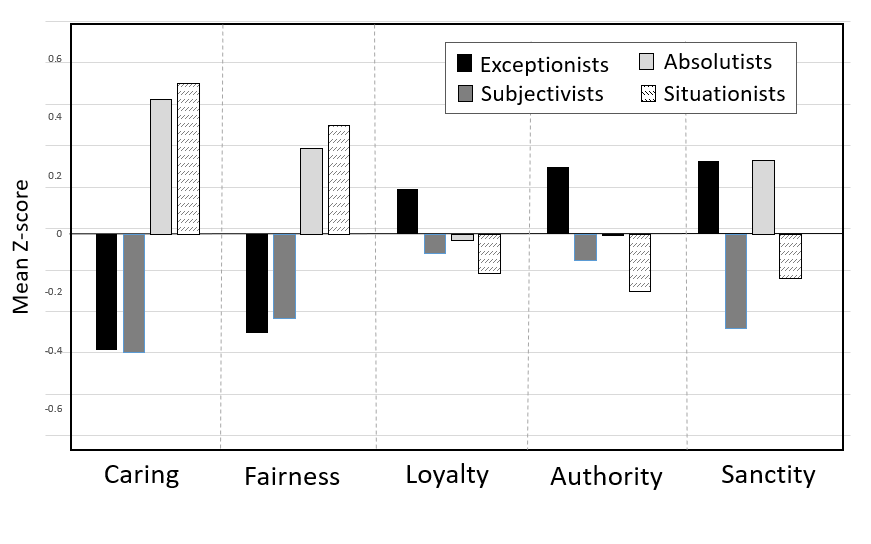

Supplement: S2 Text — (DOCX) [file pone.0251989.s004.docx]
