# Supplementary material for: Individual differences in ethics positions: The EPQ-5
Source: PLoS One. 2021 Jun 21;16(6):e0251989. doi: 10.1371/journal.pone.0251989 (PMC8216522; doi:10.1371/journal.pone.0251989)
Supplement: Attachment — Submitted filename: response letter for revision.docx [file pone.0251989.s005.docx]

**Response Letter**

Thank you for submitting your manuscript to PLOS ONE. After careful consideration, we feel that it has merit but please check the spelling.

**Authors’ reply**: Thank you very much for the opportunity to revise our manuscript! We have revised it with close attention to the issues raised by you and the reviewer. We feel the manuscript now reads much more smoothly and we’re grateful to you and the review team.

Please submit your revised manuscript by May 21 2021 11:59PM. If you will need more time than this to complete your revisions, please reply to this message or contact the journal office at [plosone@plos.org](mailto:plosone@plos.org). When you're ready to submit your revision, log on to <https://www.editorialmanager.com/pone/> and select the 'Submissions Needing Revision' folder to locate your manuscript file.

Please include the following items when submitting your revised manuscript:

- A rebuttal letter that responds to each point raised by the academic editor and reviewer(s). You should upload this letter as a separate file labeled 'Response to Reviewers'.
- A marked-up copy of your manuscript that highlights changes made to the original version. You should upload this as a separate file labeled 'Revised Manuscript with Track Changes'.
- An unmarked version of your revised paper without tracked changes. You should upload this as a separate file labeled 'Manuscript'.

**Authors’ reply**: Confirmed

If you would like to make changes to your financial disclosure, please include your updated statement in your cover letter. Guidelines for resubmitting your figure files are available below the reviewer comments at the end of this letter.

If applicable, we recommend that you deposit your laboratory protocols in protocols.io to enhance the reproducibility of your results. Protocols.io assigns your protocol its own identifier (DOI) so that it can be cited independently in the future. For instructions see: <http://journals.plos.org/plosone/s/submission-guidelines#loc-laboratory-protocols>. Additionally, PLOS ONE offers an option for publishing peer-reviewed Lab Protocol articles, which describe protocols hosted on protocols.io. Read more information on sharing protocols at <https://plos.org/protocols?utm_medium=editorial-email&utm_source=authorletters&utm_campaign=protocols>.

We look forward to receiving your revised manuscript.

**Authors’ reply**: We have now included information regarding financial support from our universities and chairs in the cover letter.

Kind regards,

Frantisek Sudzina
Academic Editor
PLOS ONE

Journal Requirements:

When submitting your revision, we need you to address these additional requirements.

1. Please ensure that your manuscript meets PLOS ONE's style requirements, including those for file naming. The PLOS ONE style templates can be found at

<https://journals.plos.org/plosone/s/file?id=wjVg/PLOSOne_formatting_sample_main_body.pdf> and

<https://journals.plos.org/plosone/s/file?id=ba62/PLOSOne_formatting_sample_title_authors_affiliations.pdf>

**Authors’ reply**: Confirmed

2. Please review your reference list to ensure that it is complete and correct. If you have cited papers that have been retracted, please include the rationale for doing so in the manuscript text, or remove these references and replace them with relevant current references. Any changes to the reference list should be mentioned in the rebuttal letter that accompanies your revised manuscript. If you need to cite a retracted article, indicate the article’s retracted status in the References list and also include a citation and full reference for the retraction notice.

 3. Thank you for stating the following financial disclosure:

'NO. The funders had no role in study design, data collection and analysis, decision to publish, or preparation of the manuscript.'

**Authors’ reply**: Confirmed

At this time, please address the following queries:

a. Please clarify the sources of funding (financial or material support) for your study. List the grants or organizations that supported your study, including funding received from your institution.
b. State what role the funders took in the study. If the funders had no role in your study, please state: “The funders had no role in study design, data collection and analysis, decision to publish, or preparation of the manuscript.”
c. If any authors received a salary from any of your funders, please state which authors and which funders.
d. If you did not receive any funding for this study, please state: “The authors received no specific funding for this work.”

Please include your amended statements within your cover letter; we will change the online submission form on your behalf.

**Authors’ reply**: We have now added that our universities and endowed chairs supported the project on the title page. We would like to add the amended statement that the funders had no role in study design, data collection and analysis, decision to publish, or preparation of the manuscript.

 4. Please include captions for your Supporting Information files at the end of your manuscript, and update any in-text citations to match accordingly. Please see our Supporting Information guidelines for more information: [http://journals.plos.org/plosone/s/supporting-information](about:blank)


**Authors’ reply**: Confirmed

Reviewers' comments:

Reviewer's Responses to Questions

**Comments to the Author**

5. Review Comments to the Author

Reviewer #1: This paper validates and replicates a shorter version of the EPQ-5, addressing issues raised with the original EPQ. I found the paper quite compelling and expect I will make use of this instrument in my own research. I have no substantial criticisms. I did catch a couple of typos: There seems to be something off with the sentence spanning from Page 19 to 20, including that the "of" should presumably be "on", and I think a "be" after "would" and "that" should be "which"? And I think the "and" in the third line from the bottom on Page 22 should be "or".

**Authors’ reply**: Thank you very much for your positive feedback on our work. We apologize for the typos and unclear writing in places. We have now carefully read the manuscript to correct the errors you pointed out as well as any others we were able to detect. Again, we greatly appreciate your feedback.

Reviewer #2: The paper makes an important contribution and the work is clearly and cogently presented, with appropriate contextualization in the scholarly literature. I have just enough familiarity with the core methodologies to be fairly confident of my 'yes' answer to Question 1, on the condition that you have an expert in psychometric analysis address Question 2, to which I answered 'I don't know' because the technicalities are outside the scope of my home discipline.

I enjoyed reading the paper and found the style accessible and pleasingly crisp.

**Authors’ reply**: Thank you very much for your support and guidance!

Typographical errors to correct:
p. 7, line 5: 'achieved' should be 'achieve';
p. 7, last line: insert 'of' between 'studies' and 'morality';
p. 20, top line: insert 'be' between 'would' and 'disinclined';
p. 23, 5th line from the bottom, remove extraneous 'The' from 'TheIn' to yield 'In contrast...';
p. 26, I believe 'tenants' should rather be 'tenets';
Other items:
p. 11, spell out abbreviation 'EPT', which occurs twice at p. 11 (or correct to 'EPQ' if that was the intended meaning);
p. 16, insert 'that' between 'predicts' and 'individuals' for easier reading;
p. 26, I would say 'a common practice' rather than 'a widely engaged in practice'

**Authors’ reply**: We again apologize for these errors. We have strived to present a fully polished manuscript for maximum readability and free of any mistakes. Thank you again!

It would be nice to see the name or at least some kind of informative description of the online survey site mentioned at p. 8.

**Authors’ reply**: Thank you for raising this issue. The site included the name of one of the authors and their affiliation. We were trying to preserve the blind review process, but admittedly we could have gone about it in a more direct way (i.e., “website blinded during peer review”). We’ve now adjusted that language and if our paper is accepted we will unmask the URL.
